# Treatment of Slaughter House Wastewater in a Sequencing Batch Reactor: Performance Evaluation and Biodegradation Kinetics

**DOI:** 10.1155/2013/134872

**Published:** 2013-08-20

**Authors:** Pradyut Kundu, Anupam Debsarkar, Somnath Mukherjee

**Affiliations:** Environmental Engineering Division, Civil Engineering Department, Jadavpur University, Kolkata 32, India

## Abstract

Slaughterhouse wastewater contains diluted blood, protein, fat, and suspended solids, as a result the organic and nutrient concentration in this wastewater is vary high and the residues are partially solubilized, leading to a highly contaminating effect in riverbeds and other water bodies if the same is let off untreated. The performance of a laboratory-scale Sequencing Batch Reactor (SBR) has been investigated in aerobic-anoxic sequential mode for simultaneous removal of organic carbon and nitrogen from slaughterhouse wastewater. The reactor was operated under three different variations of aerobic-anoxic sequence, namely, (4+4), (5+3), and (3+5) hr. of total react period with two different sets of influent soluble COD (SCOD) and ammonia nitrogen (NH_4_^+^-N) level 1000 ± 50 mg/L, and 90 ± 10 mg/L, 1000 ± 50 mg/L and 180 ± 10 mg/L, respectively. It was observed that from 86 to 95% of SCOD removal is accomplished at the end of 8.0 hr of total react period. In case of (4+4) aerobic-anoxic operating cycle, a reasonable degree of nitrification 90.12 and 74.75% corresponding to initial NH_4_^+^-N value of 96.58 and 176.85 mg/L, respectively, were achieved. The biokinetic coefficients (*k*, *K*_*s*_, *Y*, *k*_*d*_) were also determined for performance evaluation of SBR for scaling full-scale reactor in future operation.

## 1. Introduction 

The continuous drive to increase meat production for the protein needs of the ever increasing world population has some pollution problems attached. Pollution arises from activities in meat production as a result of failure in adhering to Good Manufacturing Practices (GMP) and Good Hygiene Practices (GHP) [1]. Consideration is hardly given to safety practices during animal transport to the abattoir, during slaughter and dressing of hides and flesh [2]. For hygienic reasons abattoirs, use large amount of water in processing operations (slaughtering and cleaning), which produces large amount of wastewater. The major environmental problem associated with this abattoir wastewater is the large amount of suspended solids and liquid waste as well as odor generation [3]. Effluent from slaughterhouses has also been recognized to contaminate both surface and groundwater because during abattoir processing, blood, fat, manure, urine, and meat tissues are lost to the wastewater streams [4]. Leaching into groundwater is a major part of the concern, especially due to the recalcitrant nature of some contaminants [5]. Blood, one of the major dissolved pollutants in abattoir wastewater, has the highest COD of any effluent from abattoir operations. If the blood from a single cow carcass is allowed to discharge directly into a sewer line, the effluent load would be equivalent to the total sewage produced by 50 people on average day [6]. The major characteristics of abattoir wastes are high organic strength, sufficient organic biological nutrients, adequate alkalinity, relatively high temperature (20 to 30°C) and free of toxic material. Abattoir wastewaters with the previous characteristics are well suited to anaerobic treatment and the efficiency in reducing the BOD_5_ ranged between 60 and 90% [7]. The high concentration of nitrates in the abattoir wastewater also exhibits that the wastewater could be treated by biological processes. Nitrogenous wastewater when discharged to receiving water bodies leads to undesirable problems such as algal blooms and eutrophication in addition to oxygen deficit. The dissolved oxygen level further depleted if organic carbon along with nutrient sinks into the water environment. Hence, it is very much necessary to control the discharge of combined organic carbon and nitrogen laden wastewater by means of appropriate treatment. Biological treatment has been proved to be comparatively innocuous and more energy efficient of treating wastewater if good process control could be ensured [8]. Several researchers successfully used different technologies for treatment of slaughterhouse wastewater containing organic carbon and nitrogen (COD and TKN) in laboratory and pilot scale experiment.  Table 1 had shown the previous research findings about slaughterhouse wastewater treatment by the different investigators.

Among the various biological treatment processes, sequencing batch reactor (SBR) is considered to be an improved version of activated sludge process, which operates in fill and draw mode for biological treatment of wastewater. An SBR operates in a pseudobatch mode with aeration and sludge settlement both occurring in the same tank. SBRs are operated in fill-react-settle-draw-idle period sequences. The major differences between SBR and conventional continuous-flow, activated sludge system is that the SBR tank carries out the functions of equalization, aeration, and sedimentation in a time sequence rather than in the conventional space sequence of continuous-flow systems. Sequencing batch reactors (SBRs) are advocated as one of the best available techniques (BATs) for slaughterhouse wastewater treatment [16, 17] because they are capable of removing organic carbon, nutrients, and suspended solids from wastewater in a single tank and also have low capital and operational costs. 

Biological treatment of wastewater containing organic carbon and nitrogen (COD and TKN) is also carried out in laboratory and pilot scale experiment by several researchers successfully [18–26]. Nutrients in piggery wastewater with high organic matter, nitrogen, and phosphorous content were biological removed by Obaja et al. [27] in a sequencing batch reactor (SBR) with anaerobic, aerobic, and anoxic stages. The SBR was operated with wastewater containing 1500 mg/L ammonium and 144 mg/L phosphate, a removal efficiency of 99.7% for nitrogen and 97.3% for phosphate was obtained. A full-scale SBR system was evaluated by Lo and Liao [28] to remove 82% of BOD and more than 75% of nitrogen after a cycle period of 4.6 hour from swine wastewater. Mahvi et al. [29] carried out a pilot-scale study on removal of nitrogen both from synthetic and domestic wastewater in a continuous flow SBR and obtained a total nitrogen and TKN removal of 70–80% and 85–95%, respectively. An SBR system demonstrated by Lemaire et al. [30] to high degree of biological remove of nitrogen, phosphorus, and COD to very low levels from slaughterhouse wastewater. A high degree removal of total phosphorus (98%), total nitrogen (97%), and total COD (95%) was achieved after a 6-hour cycle period. Moreover, SBRs have been successfully used to treat landfill leachate, tannery wastewater, phenolic wastewater, and various other industrial wastewaters [31–34].

In the present investigation, an attempt has been made to explore the performance efficacy of SBR technology for simultaneous removal of soluble carbonaceous organic matter and ammonia nitrogen from slaughterhouse wastewater and also to determine the biokinetic constants for carbon oxidation, nitrification, and denitrification under different combination of react periods (aerobic/anoxic).

## 2. Material and Methods

### 2.1. Seed Acclimatization for Combined Carbon Oxidation and Nitrification

The active microbial seed was cultured under ambient condition in the laboratory by inoculating 200 mL sludge as collected from an aeration pond of M/S Mokami small-scale slaughterhouse located in the village Nazira, South 24 Parganas district (West Bengal), India, to a growth propagating media composed of 500 mL dextrose solution having concentrations of 1000 mg/L, 250 mL ammonium chloride (NH_4_Cl) solution having concentration of 200 mg/L and 250 mL of nutrient solution in 3000 mL capacity cylindrical vessel. The composition of the nutrient solution in 250 mL distilled water was comprised of 60.0 mg K_2_HPO_4_, 40.0 mg KH_2_PO_4_, 500.0 mg MgSO_4_·7H_2_O, 710.0 mg FeCl_3_·6H_2_O, 0.1 mg ZnSO_4_·7H_2_O, 0.1 mg CuSO_4_·5H_2_O, 8.0 mg MnCl_2_·2H_2_O, 0.11 mg (NH_4_)_6_Mo_7_O_24_, 100.0 mg CaCl_2_·2H_2_O, 200.0 mg CoCl_2_·6H_2_O, 55.0 mg Al_2_(SO_4_)_3_·16H_2_O, 150.0 mg H_3_BO_3_. Finally 800 mL volume of distilled water was added to liquid mixture to make a volume of 2 L and the mixture was continuously aerated with intermittent feeding with dextrose solution having concentrations of 1000 mg/L and ammonium chloride (NH_4_Cl) having concentration of 200 mg/L as a carbon and nitrogen source, respectively. The acclimatization process was continued for an overall period of 90 days. The biomass growth was monitored by the magnitude of sludge volume index (SVI) and mixed liquor suspended solid (MLVSS) concentration in the reactor. pH in the reactor was maintained in the range 6.8–7.5 by adding required amount of sodium carbonate (Na_2_CO_3_) and phosphate buffer. The seed acclimatization phase was considered to be over when a steady-state condition was observed in terms of equilibrium COD and NH_4_^+^-N reduction with respect to a steady level of MLVSS concentration and SVI in the reactor.

Denitrifying seed was cultured separately in 2.0 L capacity aspirator bottle under anoxic condition. 500 gm of digested sludge obtained from the digester of a nearby sewage treatment plant (STP) was added to 1.0 L of distilled water. The solution was filtered and 250 mL of nutrient solution along with 250 mL dextrose solution as carbon source and 100 mL potassium nitrate solution (KNO_3_) as the source of nitrate nitrogen (NO_3_^−^-N) was added to it. The resulting solution was acclimatized for denitrification purpose under anoxic condition. Magnetic stirrer was provided for proper mixing of the solution. Denitrifying seed was acclimatized against a nitrate-nitrogen concentration varying from 10–90 mg/L as N, over a period of three months.

### 2.2. Experimental Procedure

The experimental work was carried out in a laboratory scale SBR, made of Perspex sheet of 6 mm thickness, having 20.0 L of effective volume. In order to assess the treatability of slaughterhouse wastewater in an SBR, the real-life wastewater samples were collected from two different locations (i) the raw (untreated) wastewater from the main collection pit and (ii) the primary treated effluent from the inlet box of aeration basin. The wastewater samples were collected 4 (four) times over the entire course of the study in 25.0 L plastic containers and stored in a refrigerator at approximately 4.0°C. The effluent quality was examined as per the methods described in “Standard Methods” [35] for determining its initial characteristics which are exhibited in Table 2. 

**Figure 1:**
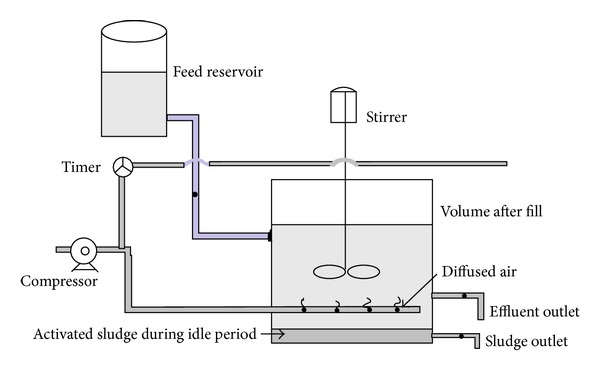
A schematic diagram of the experimental setup.

The settled effluent was poured in the reactor of 20.0 L capacity to perform necessary experiments. 2.5 L of preacclimatized mixed seed containing carbonaceous bacteria, nitrifier, and denitrifier was added in the reactor containing 20.0 L of pretreated slaughterhouse wastewater to carry out the necessary experiments. Oxygen was supplied through belt-driven small air compressor. A stirrer of 0.3 KW capacity was installed at the center of the vessel for mixing the content of the reactor. Air was supplied to the reactor during aerobic phase of react period with the help of diffused aeration system. However, during the anoxic phase the stirrer was allowed only to operate for mixing purpose and air supply was cut off. A timer was also connected to compressor for controlling the sequence of different react period (aerobic and anoxic). A schematic diagram of the experimental setup is shown in Figure 1.

The cycle period for the operation of SBR was taken as 10 hour, with a fill period of 0.5 hour, overall react period of 8.0 hours, settle period of 1.0 hour, and idle/decant period of 0.5 hour. The overall react period was divided into aerobic and anoxic react period in the following sequences:

 
*Combination-1:* 4-hour aerobic react period and 4-hour anoxic react period.
 
*Combination-2:* 5-hour aerobic react period and 3-hour anoxic react period.
 
*Combination-3:* 3-hour aerobic react period and 5-hour anoxic react period.

The performance study was carried out with pretreated slaughterhouse wastewater with same initial soluble chemical oxygen demand (SCOD) and two different ammonia nitrogen (NH_4_^+^-N) concentration of 1000 ± 50 mg/L and 90 ± 10 mg/L, 1000 ± 50 mg/L and 180 ± 10 mg/L, respectively. During the fill period of 30 min duration, 16.0 L of slaughterhouse wastewater was transferred under gravity from a feeding tank into the reactor. The mechanical mixer was operated continuously with a speed of 400 rpm from the beginning of the fill phase till the end of the total react phase for proper mixing of liquid in the reactor. During the draw phase, the supernatant wastewater was decanted until the liquid volume in the reactor was decreased to 4.0 L. sludge retention time (SRT) was manually controlled by withdrawal of volume of the mixed liquor from the reactor every day at the onset of the commencement of settle phase. The reactor was continuously run for 120 days. The initial pH values in the reactor were kept in between 7.1 and 7.7, whereas the sludge volume index (SVI) has been kept within the range of 75–85 mL/gm, for obtaining good settling property of the biomass. It has been reported that SRT should be longer than 10 days to achieve efficient nitrogen removal [36]. The SRT of 20–25 days as maintained for carbon oxidation and nitrification in the present SBR system for treatment of wastewater as suggested by Tremblay et al. [37]. 

During the time course of the study, 100 mL of sample was collected from the outlet of the reactor at every 1.0 hour interval, till completion of the fill period. The samples were analyzed for the following parameters: pH, DO, MLSS, MLVSS, COD, NH_4_^+^-N, NO_2_^−^-N, and NO_3_^−^-N as per the methods described in “Standard Methods” [35]. The pH of the solution was measured by a digital pH meter. NH_4_^+^-N, NO_2_^−^-N, and NO_3_^−^-N were estimated by respective ion selective electrodes in ISE meter. COD was analyzed by closed reflux method using dichromate digestion principle in digester. Dissolved oxygen (DO) was measured electrometrically by digital DO meter. Mixed liquor suspended solids (MLSS) and Mixed liquor volatile suspended solids (MLVSS) were measured by gravimetric method at temperature of 103–105°C and 550 ± 50°C in muffle furnace, respectively.

### 2.3. Carbon Oxidation and Nitrification Kinetics in SBR

Biokinetic parameters play an important role in designing and optimizing an activated sludge process. The biokinetic constants describe the metabolic performance of the microorganisms when subjected to the substrate and other components of the specific wastewater. These biokinetic coefficients yield a set of realistic design parameters, which can be used in rationalizing the design of the activated sludge process for a specific substrate.

#### 2.3.1. Substrate Removal Kinetics

The substrate removal constants, namely, half saturation concentration (*K*_*s*_) and the maximum rate of substrate utilization (*k*) were determined from the Lawrence and McCarty's modified Monod equation [38] given below:

(1) $$\begin{array}{c} \frac{1}{U}=\left(\frac{K_{s}}{k}\right)\left(\frac{1}{S}\right)+\frac{1}{k} \end{array}$$

*S* = Substrate (SCOD and NH_4_^+^-N) concentration at any time in reactor (mg/L), *U* = Specific substrate utilization rate = (*S*_0_ − *S*)/*θX* (mg of SCOD or mg of NH_4_^+^-N/day/mg of MLVSS), *θ* = Contact  time (day), *X* = MLVSS at any time in the reactor (mg/L), *S*_0_ = Substrate (SCOD and NH_4_^+^-N) concentration of the influent (mg/L).

The plots made between 1/*U* and 1/*S* develops into a straight line with *K*_*s*_/*k* as its slope and 1/*k* as its intercept. 

#### 2.3.2. Sludge Growth Kinetics

The sludge growth kinetic constants namely the yield coefficient (*Y*) and the endogenous decay coefficient (*K*_*d*_), were determined from the Lawrence and McCarty's modified Monod equation [38] given below:

(2) $$\begin{array}{c} \frac{1}{\theta }=YU-K_{d}, \end{array}$$

where *U* = Specific substrate utilization rate (mg of SCOD or mg of NH_4_^+^-N/day/mg of MLVSS), *θ* = Contact time (day), *k*_*d*_ = Endogenous decay coefficient (day^−1^), and *Y* = Yield coefficient (mg of MLVSS produced/mg of SCOD or NH_4_^+^-N).

A graph drawn between 1/*θ* and *U* gives a straight line, with *Y* as its slope and *k*_*d*_ as its intercept.

### 2.4. Denitrification Kinetics in SBR

In almost all cases denitrification started occurring at the onset of anoxic period and specific denitrification rate (*q*_DN_) was calculated under different initial organic carbon and NH_4_^+^-N concentrations for different react period combinations, namely, (4+4), (5+3), (3+5) hrs over the respective anoxic environment. 

## 3. Results and Discussion

### 3.1. Carbon Oxidation Performance

**Figure 2:**
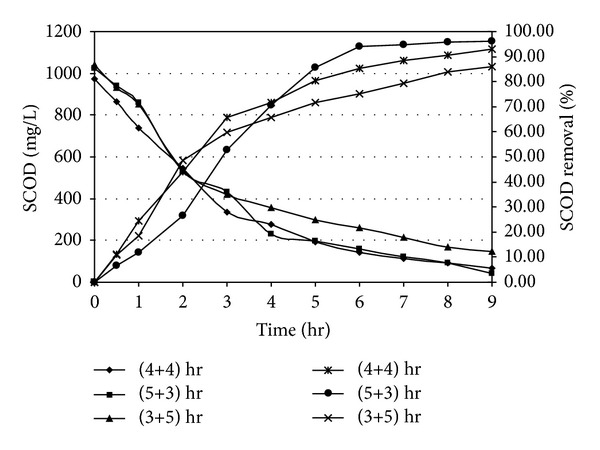
Carbon oxidation profile under different react period combination [Initial SCOD = 1000 ± 50 mg/L; Initial NH_4_^+^-N = 90 ± 10 mg/L as N].

**Figure 3:**
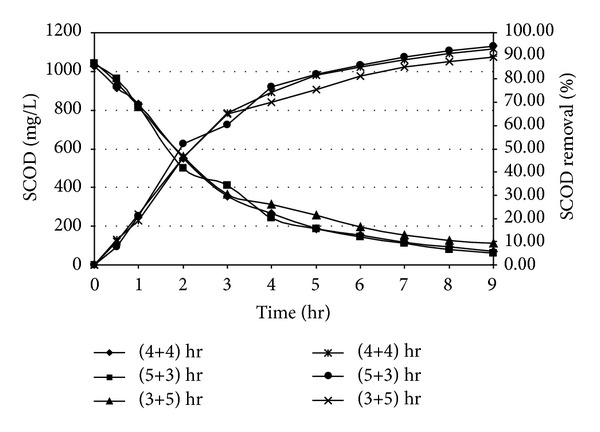
Carbon oxidation profile in SBR under different react period combination [Initial SCOD = 1000 ± 50 mg/L; Initial NH_4_^+^-N = 180 ± 10 mg/L as N].

Organic carbon, which is the source of energy for heterogenic and denitrifying microorganism, was estimated as chemical oxygen demand (COD). In the present experiment, in correspondance to an initial SCOD of 975.25 mg/L and initial NH_4_^+^-N concentration of 87.52 mg/L as N, it has been observed that the major fraction of SCOD removal took place within 4 or 5 hrs of aerobic react period. In anoxic phase, further SCOD removal has been noticed as shown in Figure 2. Li et al. [39] obtained that the maximum removal efficiency of COD (96%) for treatment of slaughterhouse wastewater which was marginally higher than the result of this present study. COD removal profile was also observed in similar pattern in the presence of higher initial NH_4_^+^-N concentration of 185.24 mg/L as N and initial SCOD of 1028.55 mg/L in a separate set of experiment. The results are plotted in Figure 3. It is revealed from Figures 2 and 3 that the rate of organics utilization by the dominant heterotrophs during initial aerobic react period was more as compared to its rate of removal during anoxic period. The carbon utilization bacteria used up bulk amount COD for energy requirement and growth. The removal efficiency of COD in the suspended growth reactor system depends on COD : TKN ratio. The mean COD : TKN ratio recommended for adequate carbon oxidation and nutrient removal as 10–12 [40]. In the present investigation, COD : TKN ratio was approximately 11.14 which was in agreement in their recommendation. The removal efficiency also depends on react time. The carbon utilizing bacteria obviously and is able to degrade more COD and produce CO_2_ with production of new cells due to enhancement of aeration time. A marked improvement has been noticed for higher percentage removal of COD during increase of aeration time. A similar observation was noticed by Kanimozhi and Vasudevan [41]. Due to the increase of time and COD load more cells to be produced eventually higher degree of organic removal. When the react period was changed into 5-hour aerobic followed by a reduced 3.0 hour anoxic, a marginal improvement of SCOD removal in aerobic phase (77.27%) and anoxic phase (96.07%) with an initial SCOD of 1023.22 mg/L was observed due to enhanced aeration time. On the other hand, when the react period was subsequently changed to 3.0 hour, aerobic period followed by 5.0-hour anoxic period, a marginal decrease of SCOD removal in aerobic phase (65.64%) and anoxic phase (86.07%) with an initial SCOD of 1042.52 mg/L was obtained due to lag of aeration time. 

### 3.2. Nitrification Performance

**Figure 4:**
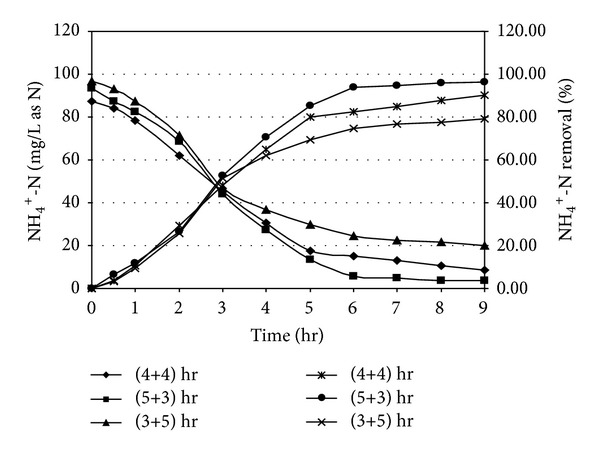
Ammonia oxidation profile in SBR under different react period combination [Initial SCOD = 1000 ± 50 mg/L; Initial NH_4_^+^-N = 90 ± 10 mg/L as N].

**Figure 5:**
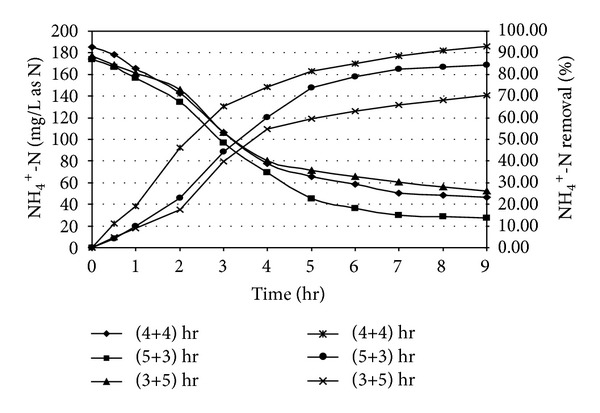
Ammonia oxidation profile in SBR under different react period combination [Initial SCOD = 1000 ± 50 mg/L; Initial NH_4_^+^-N = 180 ± 10 mg/L as N].

Ammonia oxidation took place due to the presence of previously acclimatized nitrifying organisms within the reactor as mixed culture. The nitrification results are shown in Figures 4 and 5. In case of specific cycle period of 4 hr (aerobic) and 4 hr (anoxic), it was observed that at the end of 8 hr react period of reaction, 90.12% nitrification could achieved for an initial NH_4_^+^-N was approximately 87.52 mg/L as Fongsatitkul et al. [40] obtained maximum 93% removal efficiency of soluble nitrogen for treatment of abattoir wastewater which was slightly higher than our result. The ammonia oxidation occurred in two phases; a fraction of ammonia was assimilated by cell-mass for synthesis of new cell during carbon oxidation and, in the subsequent phase, dissimilatory ammonia removal took place for converting NH_4_^+^-N into NO_2_^−^-N and NO_3_^−^-N under aerobic period. The dissimilatory removal of ammonia depends on the population of nitrifiers and oxidation time. The descending trend of ammonia removal for higher level of initial concentration of NH_4_^+^-N was attributed due to limitation of enzymatic metabolism of nitrifiers. When the reactor system was operated in 5 hr (aerobic) and 3 hr (anoxic) mode of react cycle, an overall performance of ammonia oxidation was improved from 90.12 to 96.20% and 84.41% for initial NH_4_^+^-N of 93.54 mg/L and 173.88 mg/L as N, respectively. Higher oxidation period was also recommended by earlier investigators [42, 43] for describing similar kind of experiment on landfill leachate treatment in SBR. The results reveal the fact that the extension of aeration period helped to enhance the oxidation efficiency for the present system. It was also observed that when aerobic period was reduced to 3.0 hr, ammonia oxidation reduced to 79.18% and 70.53% corresponding to initial NH_4_^+^-N value of 96.58 mg/L and 176.85 mg/L, respectively, at the end of 8 hr react period. 

### 3.3. Denitrification Performance

**Figure 6:**
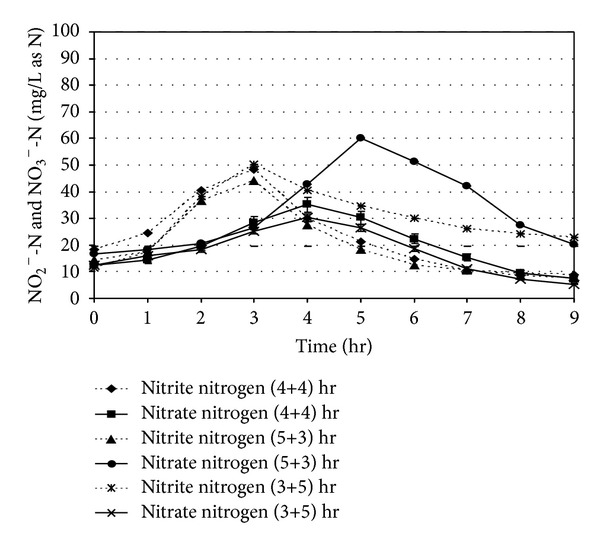
Nitrite and nitrate concentration profiles in SBR under different react period combination [initial SCOD = 1000 ± 50 mg/L and initial NH_4_^+^-N = 90 ± 10 mg/L as N].

The nitrite and nitrate nitrogen (NO_2_^−^-N and NO_3_^−^-N) level in the reactor during the total reaction period is shown in Figure 6. The maximum nitrite level was observed in between 2.5 and 3.0 hr of react period. The peak nitrate (NO_3_^−^) level was found to be formed close to 4.0 hr of aeration period for (4+4) and (3+5) hr combinations of react period. A time lag of one hour for maximum nitrate formation was also noticed after the attainment of the maximum NO_2_^−^-N level in the reactor. For (5+3) hr react period combination, the formation of NO_3_^−^ showed a time-dependent factor as the peak was found at the end of 5.0 hrs. In the Figure 6, after 4.0 hr of aeration period, the NO_3_^−^ level was found to be 35.21 mg/L as N corresponding to initial NH_4_^+^-N level of 87.52 mg/L as N and NO_3_^−^ concentration of 12.35 mg/L as N, respectively. On the other hand, after 5.0 hour of aerated react period, NO_3_^−^-N concentration in the reactor was found to be 60.24 mg/L as N for an initial NH_4_^+^-N and NO_3_^−^-N concentration of 93.54 and 16.52 mg/L as N, respectively. The maximum NO_3_^−^-N concentration for (3+5) hour react period combination was found to be 25.31 mg/L as N for the initial NH_4_^+^-N concentration of 96.58 mg/L as N and NO_3_^−^-N level of 12.35 mg/L as N. The experimental results clearly indicate the necessity of longer aeration period for achieving maximum utilization of ammonia by the nitrifiers. 

**Figure 7:**
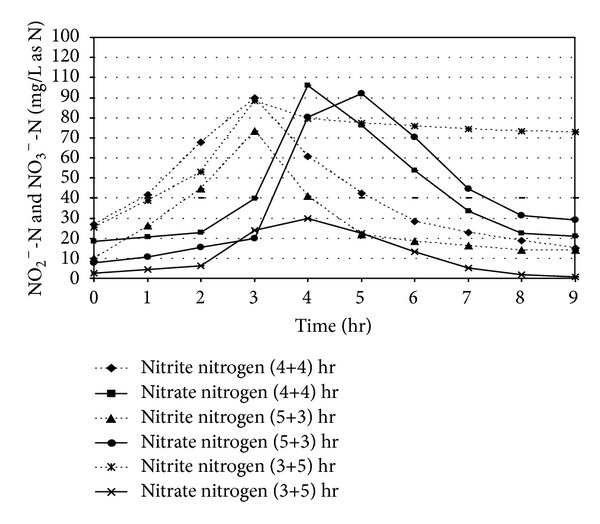
Nitrite and nitrate concentration profiles in SBR under different react period combination [initial SCOD = 1000 ± 50 mg/L and initial NH_4_^+^-N = 180 ± 10 mg/L as N].

 In Figure 7, after 4.0 hour of anoxic react period, nitrate (NO_3_^−^) was reduced to 22.29 mg/L as N from its peak concentration of 96.22 mg/L as N, which achieved a 76.83% removal of nitrate for initial NH_4_^+^-N concentration 185.24 mg/L as N. During denitrification phase, the residual soluble COD concentration as available was found to be more than the stoichiometric organic carbon requirement for effective denitrification. When the anoxic react period was reduced to 3.0 hr, it was observed that, nitrate concentration after 5 hr of aerobic period was found to be maximum (92.11 mg/L as N), per cent removal of nitrate descended from 76.83 to 66.16% for initial NH_4_^+^-N concentration 173.88 mg/L as N, due to insufficient of anoxic period. 

### 3.4. MLVSS, pH, Alkalinity, and DO Profiles in the SBR during Experiment

**Figure 8:**
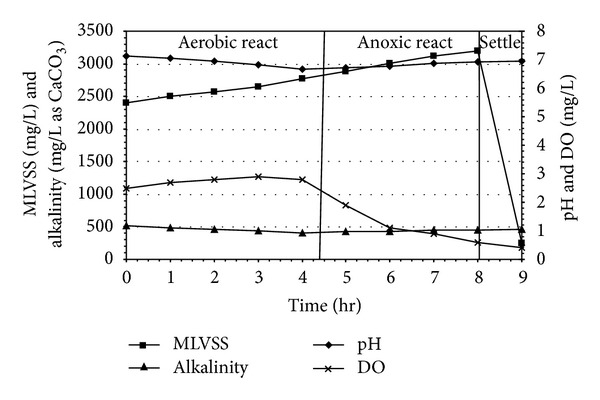
MLVSS, pH, alkalinity, and DO profiles for slaughterhouse wastewater treatment in SBR under (4+4) hr react period combination.

**Figure 9:**
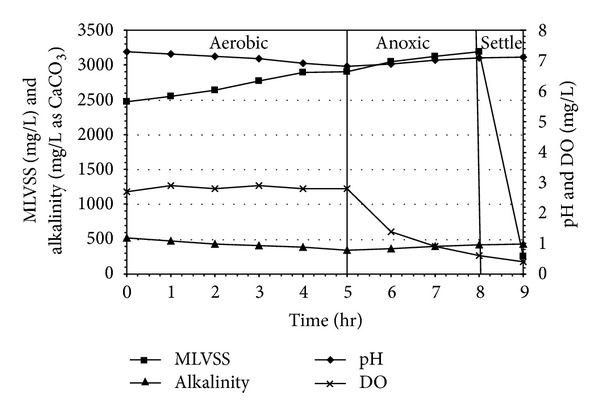
MLVSS, pH, alkalinity, and DO profiles for slaughterhouse wastewater treatment in SBR under (5+3) hr react period combination.

**Figure 10:**
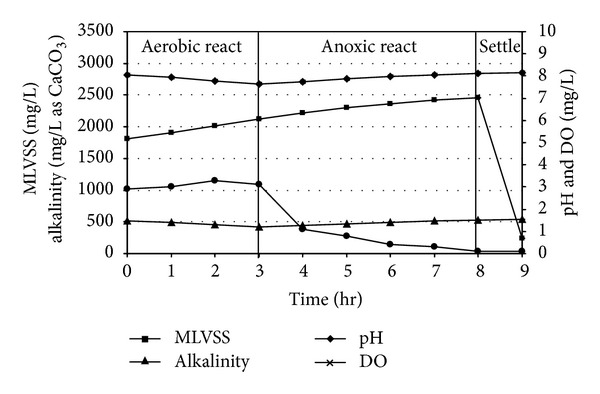
MLVSS, pH, alkalinity, and DO profiles for slaughterhouse wastewater treatment in SBR under (3+5) hr react period combination.

The pH and alkalinity values of a biological system are vital parameters for microbial denitrification. The value of pH increases for ammonification and denitrification, decreases for organic carbon oxidation and nitrification. Alkalinity is not only important for nitrification and denitrification, but to also be used for indicating the system stability. Alkalinity was found to have a close correlation with SBR operating conditions, since different extents of nitrification (alkalinity consumption) and denitrification (alkalinity production) contribute to the variation of alkalinity in the system. During the aerobic phase, the minimal value of the pH curve was characterized the end of nitrification (Figures 8, 9, and 10). At the beginning of anoxic react phase, when ammonia nitrogen concentration was reduced considerably, pH starts to increase. This has occurred between 4.0 and 5.0 hr after the starting of aerobic react period in all experimental sets. The DO profile exhibited a sharp fall after which DO concentration decreased markedly at anoxic phase and reached minimum value. In the present, study the DO level remained almost steady during the entire aerobic react period with a marginal increase in DO level, but a marked descending trend was observed during the anoxic period in all the reaction sets irrespective of initial SCOD and ammonia concentrations. Under strict anaerobic condition the DO should be equal to zero, but anoxic environment starts from DO level less than 1.5 mg/L. At the start of anoxic react period most of the cases, DO was found to be less than 1.5 mg/L and at the end of anoxic react period the value becomes less than 1.0 mg/L.

### 3.5. Kinetic Study for Organic Carbon Removal from Slaughterhouse Wastewater in SBR

In the present study, the performance evaluation of the SBR system was also carried out from the view point of reaction kinetics determination for treating slaughterhouse wastewater. The values for the reciprocal of specific substrate utilization rate (1/*U*) were plotted against the reciprocal of effluent SCOD (1/*S*) and substrate removal kinetics was evaluated using (1) as stated earlier. The slope of the straight line is (*K*_*s*_/*k*) and intercept is (1/*k*). The reciprocal of the contact time (1/*θ*) were plotted against the specific substrate utilization rate (*U*) and microbial growth kinetics was evaluated using (2). The yield coefficient (*Y*) was determined from the slope of the line and the endogenous decay coefficient (*k*_*d*_) was obtained from intercept, *k*_*d*_ = −*C*. The values of biokinetic coefficients (*k*, *K*_*s*_, *Y*, *k*_*d*_) for combined carbon-oxidation and nitrification are listed in Table 3.

From Table 3, it has been estimated that the value of yield coefficient (*Y*) for the heterotrophs is in the range from 0.485 to 0.622. The yield coefficient was found to be improved with the increase in aeration period. The half velocity constant (*K*_*s*_) values were found in the range of 149.64 to 364.81 for different combinations of react period. In the case of (5+3) combination of react cycle, the *k* and *Y* values are marginally higher than (4+4) and (3+5) combination. It was attributed to the fact that, after the initial acclimatization; the heterotrophs converted the carbon content at 5.0 hrs period of time more efficiently. After 5.0 hrs of aerobic react period, the available carbon content was reduced considerably and a fraction of heterotrophs attained endogenous state of condition while the nitrifiers are rejuvenated and started nitrification activity. This metabolism is also supported by the value of endogenous decay rate constant (*k*_*d*_). In the case of (5+3) combination of react cycle *k*_*d*_ value is found to be 0.057 which is between 0.051 and 0.047 for the cases of (4+4) and (3+5) react period combinations, respectively. The values of biokinetic coefficients, other than *K*_*s*_, such as *k*, *Y*, *k*_*d*_ as obtained from the test results for carbon-oxidation and nitrification are also in congruence with their respective typical values [44].

### 3.6. Kinetic Study for Ammonium Nitrogen Removal from Slaughterhouse Wastewater in SBR

The nitrification removal kinetics for mixed population (heterotrophs and nitrifiers) followed an identical pattern to organic carbon removal kinetics. A fraction of biological oxidation was attributed to the fact that a mixed population performed in the reactor along with nitrifiers. The linear graphs are plotted between (1/*S*) and (1/*U*) for substrate utilization kinetics under three different combinations of react period, namely, (4+4), (5+3), and (3+5), respectively, using (1). Microbial growth kinetics was evaluated using (2), which were determined by plotting straight lines between (1/*θ*) and (*U*) under three different combinations of react period, namely, (4+4), (5+3) and (3+5) hrs, respectively. The kinetic coefficient values for nitrification from the previous plots are given in Table 4. It has been clearly shown earlier that an increasing trend of higher removal efficiency for ammonia oxidation could be observed for extension of the aerobic react period beyond 4 hrs. This previous phenomenon also reflected the magnitudes of biokinetic constants under all experimental combinations of react period. The kinetic coefficients *Y*, *k*_*d*_, and *K*_*s*_ were found to be in the range of 0.205 to 0.284, 0.037 to 0.051, and 21.83 to 70.93, respectively. The ammonia concentration found in the slaughterhouse wastewater was very high 180 ± 10 mg/L as N for an inlet SCOD concentration of 1000 ± 50 mg/L, which are not usually present in any municipal wastewater stream. For this reason, the *K*_*s*_ value was found to be higher than the standard values (0.2–5.0 mg/L) considered for nitrification of municipal wastewater stream [44]. 

### 3.7. Denitrification Rates for Treatment of Slaughterhouse Wastewater in SBR

Specific denitrification rate (*q*_DN_) was measured in terms of the rate of NO_3_^−^-N removed per unit mass of denitrifying microorganisms, for three different react period combinations, namely, (4+4), (5+3), and (3+5) under the respective anoxic environment and the results are listed in Table 5. The specific denitrification rate (*q*_DN_) is expressed on average basis spanningover respective anoxic periods of 3.0, 4.0, and 5.0 hours. The average specific denitrification rate (*q*_DN_), in (5+3), (4+4), and (3+5) cases was found to increase considerably with the increase in average anoxic SCOD utilization rate (*q*_SCOD_) when primary treated effluent was considered for treatment in present SBR system. Average specific denitrification rate (*q*_DN_) varied from 4.64 to 5.42 mg of N/gm MLVSS. hr for primary treated slaughterhouse wastewater for 3 hr anoxic period. The average 4.0 hourly specific denitrification rate (*q*_DN_) varied from 4.95 to 5.88 mg of N/gm MLVSS. hr. The previous rate of specific denitrification rate (*q*_DN_) was found to be followed in similar results as reported by Barnes and Bliss [45].

## 4. Conclusions

The present experimental investigation demonstrated that sequential batch reactor (SBR) is a variable and efficient biological method to treat slaughterhouse wastewater in a single unit. The total react period of 8 hr (4 hr aerobic and 4 hr anoxic) yielded optimum carbon oxidation, nitrification, and denitrification for treatment of carbonaceous and nitrogenous wastewater. The increase in MLVSS level in the reactor exhibited the growth favoring environment of the microorganism. The pH level in the SBR descended initially during aerobic period due to nitrification and carbon oxidation followed by an increasing trend indicating the existence of denitrifiers. This phenomenon has also been established by the variation of alkalinity level during aerobic and anoxic react period. The estimated values of biokinetic coefficients (*k*, *K*_*s*_, *Y*, *k*_*d*_) showed reasonable agreement with the literature values. The kinetic data and rate reaction constants could be used for the design of a field scale SBR for treating slaughterhouse wastewater. A design rationale can be evaluated on the basis of present experimental data for the purpose of application of this technology in similar plants. The outcome of the present investigation results would be helpful for making a design rationale for SBR treatment of slaughterhouse wastewater and a pilot plant study can be conducted with real-life wastewater sample by application of derived data of present study. In the future scope of the study, microbial genomics study including phosphate removal aspects would be also considered. The influence of solid retention time (SRT) should be explored also. A real-time kinetics profile with automatic data plotting could be derived for explaining the process in more rational way. It is also suggested that optimization of the process and operation variable may be examined with soft computing tools using various statistical approach.

## Figures and Tables

**Table 1:** Different available technologies used to treat slaughterhouse wastewater.

Sl no.	Technology adopted	Input characteristics of slaughterhouse wastewater	Observations	References
(1)	Anaerobic treatment of slaughterhouse wastewaters in a UASB (Upflow Anaerobic Sludge Blanket) reactor and in an anaerobic filter (AF).	Slaughterhouse wastewater showed the highest organic content with an average COD of 8000 mg/L, of which 70% was proteins. The suspended solids content represented between 15 and 30% of the COD.	The UASB reactor was run at OLR (Organic Loading Rates) of 1–6.5 kg COD/m^3^/day. The COD removal was 90% for OLR up to 5 kg COD/m^3^/day and 60% for an OLR of 6.5 kg COD/m^3^/day. For similar organic loading rates, the AF showed lower removal efficiencies and lower percentages of methanization.	Ruiz et al. [9]

(2)	Anaerobic sequencing batch reactors.	Influent total chemical oxygen demand (TCOD) ranged from 6908 to 11 500 mg/L, of which approximately 50% were in the form of suspended solids (SS).	Total COD was reduced by 90% to 96% at organic loading rates (OLRs) ranging from 2.07 to 4.93 kg m^−3^ d^−1^ and a hydraulic retention time of 2 days. Soluble COD was reduced by over 95% in most samples.	Massé and Masse [10]

(3)	Moving bed sequencing batch reactor for piggery wastewater treatment.	COD, BOD, and suspended solids in the range of 4700–5900 mg/L, 1500–2300 mg/L, and 4000–8000 mg/L, respectively.	COD and BOD removal efficiency was greater than 80% and 90%, respectively at high organic loads of 1.18–2.36 kg COD/m^3^·d. The moving-bed SBR gave TKN removal efficiency of 86–93%.	Sombatsompop et al. [11]

(4)	Fixed bed sequencing batch reactor (FBSBR).	The wastewater has COD loadings in the range of 0.5–1.5 Kg COD/m^3^ per day.	COD, TN, and phosphorus removal efficiencies were at range of 90–96%, 60–88%, and 76–90%, respectively.	Rahimi et al. [12]

(5)	Chemical coagulation and electrocoagulation techniques.	COD and BOD_5_ of raw wastewater in the range of 5817 ± 473 and 2543 ± 362 mg/L.	Removal of COD and BOD_5_ more than 99% was obtained by adding 100 mg/L PACl and applied voltage 40 V.	Bazrafshan et al. [13]

(6)	Hybrid upflow anaerobic sludge blanket (HUASB) reactor for treating poultry slaughterhouse wastewater.	Slaughterhouse wastewater showed total COD 3000–4800 mg/L, soluble COD 1030–3000 mg/L, BOD5 750–1890 mg/L, suspended solids 300–950 mg/L, alkalinity (as CaCO_3_) 600–1340 mg/L, VFA (as acetate) 250–540 mg/L, and pH 7–7.6.	The HUSB reactor was run at OLD of 19 kg COD/m^3^/day and achieved TCOD and SCOD removal efficiencies of 70–86% and 80–92%, respectively. The biogas was varied between 1.1 and 5.2 m^3^/m^3^ d with the maximum methane content of 72%.	Rajakumar et al. [14]

(7)	Anaerobic hybrid reactor was packed with light weight floating media.	COD, BOD and Suspended Solids in the range of 22000–27500 mg/L, 10800–14600 mg/L, and 1280–1500 mg/L, respectively.	COD and BOD reduction was found in the range of 86.0–93.58% and 88.9–95.71%, respectively.	Sunder and Satyanarayan [15]

**Table 2:** Characteristics and composition of slaughterhouse wastewater.

Parameters	Raw wastewater					Pretreated wastewater				
	Set-1	Set-2	Set-3	Set-4	Range	Set-1	Set-2	Set-3	Set-4	Range
pH	8.0	8.2	8.5	8.4	8.0–8.5	7.5	7.2	8.5	7.8	7.5–8.5
TSS (mg/L)	10120	12565	14225	13355	10120–14225	2055	2280	2540	2386	2055–2540
TDS (mg/L)	6345	7056	7840	6865	6345–7840	2800	3065	3230	3185	2800–3230
DO (mg/L)	0.8	1.1	0.9	1.3	0.8–1.3	1.2	1.4	1.5	1.6	1.2–1.6
SCOD (mg/L)	6185	6525	6840	6455	6185–6840	830	945	1045	925	830–1045
BOD_5_ at 20°C (mg/L)	3000	3200	3500	3350	3000–3500	210	240	265	252	210–265
TKN (mg/L as N)	1050	1130	1200	1165	1050–1200	305	420	525	485	305–525
NH_4_^+^ N (mg/L as N)	650	695	735	710	650–735	95	155	191	125	95–191

**Table 3:** Evaluation of biokinetic coefficients for carbon oxidation from slaughterhouse wastewater in SBR.

Initial SCOD (mg/L)	(4+4) hr react period combination	(5+3) hr react period combination	(3+5) hr react period combination	Standard values for kinetic constants [44]
1000 ± 50	(i) Substrate utilization- *y* = 70.32*x* + 0.215 (ii) Microbial growth- *y* = 0.522*x* − 0.051	(i) Substrate utilization- *y* = 68.22*x* + 0.187 (ii) Microbial growth- *y* = 0.622*x* − 0.057	(i) Substrate utilization- *y* = 42.65*x* + 0.285 (ii) Microbial growth- *y* = 0.485*x* − 0.047	*K* (day^−1^) = (2–10) *K*_*s*_ (mg/L SCOD) = (15–70) *Y* (mg VSS/mg SCOD) = (0.4–0.8) *k*_*d*_ (day^−1^) = (0.025–0.075)
	*Kinetic constants*: *k* (day^−1^) = 4.65 *K*_*s*_ (mg/L SCOD) = 327.06 *Y* (mg VSS/mg SCOD) = 0.522 *k*_*d*_ (day^−1^) = 0.051	*Kinetic constants*: *k* (day^−1^) = 5.34 *K*_*s*_ (mg/L SCOD) = 364.81 *Y* (mg VSS/mg SCOD) = 0.622 *k*_*d*_ (day^−1^) = 0.057	*Kinetic constants*: *k* (day^−1^) = 3.50 *K*_*s*_ (mg/L SCOD) = 149.64 *Y* (mg VSS/mg SCOD) = 0.485 *k*_*d*_ (day^−1^) = 0.047	

**Table 4:** Evaluation of biokinetic coefficients for nitrification from slaughterhouse wastewater in SBR.

Initial NH_4_^+^-N (mg/L as N)	(4+4) hr react period combination	(5+3) hr react period combination	(3+5) hr react period combination	Standard values for kinetic constants [44]
180 ± 10	(i) Substrate utilization- *y* = 2.371*x* + 0.047 (ii) Microbial growth- *y* = 0.234*x* − 0.047 *k* (day^−1^) = 21.27 *K*_*s*_ (mg/L NH_4_^+^-N) = 50.44 *Y* (mg VSS/mg NH_4_^+^-N) = 0.234 *k*_*d*_ (day^−1^) = 0.047	(i) Substrate utilization- *y* = 2.412*x* + 0.034 (ii) Microbial growth- *y* = 0.284*x* − 0.051 *k* (day^−1^) = 29.41 *K*_*s*_ (mg/L NH_4_^+^-N) = 70.93 *Y* (mg VSS/mg NH_4_^+^-N) = 0.284 *k*_*d*_ (day^−1^) = 0.051	(i) Substrate utilization- *y* = 1.223*x* + 0.056 (ii) Microbial growth- *y* = 0.205*x* − 0.037 *k* (day^−1^) = 17.85 *K*_*s*_ (mg/L NH_4_^+^-N) = 21.83 *Y* (mg VSS/mg NH_4_^+^-N) = 0.205 *k*_*d*_ (day^−1^) = 0.037	*k* (day^−1^) = (1–30) *K*_*s*_ (mg/L NH_4_^+^-N) = (0.2–5.0) *Y* (mg VSS/mg NH_4_^+^-N) = (0.1–0.3) *k*_*d*_ (day^−1^) = (0.03–0.06)

**Table 5:** Denitrification rates during anoxic react phase for treatment of slaughterhouse wastewater in SBR.

Initial NH_4_^+^-N (mg/L as N)	Initial SCOD (mg/L)	React period combination (Aerobic/Anoxic)	Avg. anoxic SCOD utilization rate (*q*_SCOD_) (mg SCOD/gm MLVSS. hr)	Specific denitrification rate (*q*_DN_) (mg N/gm MLVSS. hr)							
				1.0 hr	2.0 hr	3.0 hr	4.0 hr	5.0 hr	Avg. (3.0 hrly)	Avg. (4.0 hrly)	Avg. (5.0 hrly)
185.24	1028.55	(4+4)	26.25	4.49	5.57	5.85	3.89	—	5.30	4.95	—
173.88	1023.22	(5+3)	34.87	4.27	5.51	4.16	—	—	4.64	—	—
176.85	1042.52	(3+5)	38.15	4.57	5.55	6.16	7.24	6.23	5.42	5.88	5.95

## References

[1] Akinro AO, Ologunagba IB, Yahaya O (2009). Environmental implications of unhygienic operation of a city abattoir in Akure, Western Nigeria. *ARPN Journal of Engineering and Applied Sciences*.

[2] Singh VP, Neelam S (2011). A survey report on impact of abattoir activities management on environments. *Indian Journal of Veterinarians*.

[3] Gauri SM (2006). Treatment of wastewater from abattoirs before land application: a review. *Bioresource Technology*.

[4] Bello YO, Oyedemi DTA (2009). Impact of abattoir activities and management in residential neighbourhoods: a case study of Ogbomoso, Nigeria. *Journal of Social Science*.

[5] Muhirwa D, Nhapi I, Wali U, Banadda N, Kashaigili J, Kimwaga R (2010). Characterization of wastewater from an abattoir in Rwanda and the impact on downstream water quality. *International Journal of Ecology, Development*.

[6] Aniebo AO, Wekhe SN, Okoli IC (2009). Abattoir blood waste generation in rivers state and its environmental implications in the Niger Delta. *Toxicological and Environmental Chemistry*.

[7] Chukwu O (2008). Analysis of groundwater pollution from abattoir Waste in Minna, Nigeria. *Research Journal of Diary Science*.

[8] Grady J, Daigger G, Lim H (1999). *Biological Wastewater Treatment*.

[9] Ruiz I, Veiga MC, de Santiago P, Blfizquez R (1997). Treatment of slaughterhouse wastewater in a UASB reactor and an anaerobic filter. *Bioresource Technology*.

[10] Massé DI, Masse L (2000). Treatment of slaughterhouse wastewater in anaerobic sequencing batch reactors. *Canadian Agricultural Engineering*.

[11] Sombatsompop K, Songpim A, Reabroi S, Inkong-ngam P (2011). A comparative study of sequencing batch reactor and movingbed sequencing batch reactor for piggery wastewater treatment. *Maejo International Journal of Science and Technology*.

[12] Rahimi Y, Torabian A, Mehrdadi N, Shahmoradi B (2011). Simultaneous nitrification-denitrification and phosphorus removal in a fixed bed sequencing batch reactor (FBSBR). *Journal of Hazardous Materials*.

[13] Bazrafshan E, Mostafapour FK, Farzadkia M, Ownagh KA, Mahvi AH (2012). Slaughterhouse wastewater treatment by combined chemical coagulation and electrocoagulation process. *PLOS ONE*.

[14] Rajakumar R, Meenambal T, Saravanan PM, Ananthanarayanan P (2012). Treatment of poultry slaughterhouse wastewater in hybrid upflow anaerobic sludge blanket reactor packed with pleated poly vinyl chloride rings. *Bioresource Technology*.

[15] Sunder GC, Satyanarayan S (2013). Efficient treatment of slaughter house wastewater by anaerobic hybrid reactor packed with special floating media. *International Journal of Chemical and Physical Sciences*.

[16] European Commission (2005). *Integrated Pollution Prevention and Control: Reference Document on Best Available Techniques in the Slaughterhouses and Animal by-Products Industries*.

[17] Mahvi AH (2008). Sequencing batch reactor: a promising technology in wastewater treatment. *Iranian Journal of Environmental Health Science and Engineering*.

[18] Al-Mutairi NZ, Hamoda MF, Al-Ghusain IA (2007). Slaughterhouse wastewater treatment using date seeds as adsorbent. *Journal of Environment Science and Health*.

[19] Boopathy R, Bonvillain C, Fontenot Q, Kilgen M (2007). Biological treatment of low-salinity shrimp aquaculture wastewater using sequencing batch reactor. *International Biodeterioration and Biodegradation*.

[20] Kim HS, Choung YK, Ahn SJ, Oh HS (2008). Enhancing nitrogen removal of piggery wastewater by membrane bioreactor combined with nitrification reactor. *Desalination*.

[21] Al-Mutairi NZ, Al-Sharifi FA, Al-Shammari SB (2008). Evaluation study of a slaughterhouse wastewater treatment plant including contact-assisted activated sludge and DAF. *Desalination*.

[22] Roy D, Hassan K, Boopathy R (2010). Effect of carbon to nitrogen (C : N) ratio on nitrogen removal from shrimp production waste water using sequencing batch reactor. *Journal of Industrial Microbiology and Biotechnology*.

[23] Rajagopal R, Rousseau N, Bernet N, Béline F (2011). Combined anaerobic and activated sludge anoxic/oxic treatment for piggery wastewater. *Bioresource Technology*.

[24] Palatsi J, Vinas M, Guivernau M, Fernandez B, Flotats X (2011). Anaerobic digestion of slaughterhouse waste: main process limitations and microbial community interactions. *Bioresource Technology*.

[25] Kern C, Boopathy R (2012). Use of sequencing batch reactor in the treatment of shrimp aquaculture wastewater. *Journal of Water Sustainability*.

[26] Wang F, Liu Y, Wang J, Zhang Y, Yang H (2012). Influence of growth manner on nitrifying bacterial communities and nitrification kinetics in three lab-scale bioreactors. *Journal of Industrial Microbiology and Biotechnology*.

[27] Obaja D, Mac S, Mata-Alvarez J (2005). Biological nutrient removal by a sequencing batch reactor (SBR) using an internal organic carbon source in digested piggery wastewater. *Bioresource Technology*.

[28] Lo KV, Liao PH (2007). A full-scale sequencing batch reactor system for swine wastewater treatment. *Journal of Environmental Science and Health*.

[29] Mahvi AH, Mesdaghinia AR, Karakani F (2004). Nitrogen removal from wastewater in a continuous flow sequencing batch reactor. *Pakistan Journal of Biological Sciences*.

[30] Lemaire R, Yuan Z, Nicolas B, Marcelino M, Yilmaz G, Keller J (2009). A sequencing batch reactor system for high-level biological nitrogen and phosphorus removal from abattoir wastewater. *Biodegradation*.

[31] Aziz SQ, Aziz HA, Yusoff MS, Bashir MJK (2011). Landfill leachate treatment using powdered activated carbon augmented sequencing batch reactor (SBR) process: optimization by response surface methodology. *Journal of Hazardous Materials*.

[32] Durai G, Rajamohan N, Karthikeyan C, Rajasimman M (2010). Kinetics studies on biological treatment of tannery wastewater using mixed culture. *International Journal of Chemical and Biological Engineering*.

[33] Faouzi M, Merzouki M, Benlemlih M (2013). Contribution to optimize the biological treatment of synthetic tannery effluent by the sequencing batch reactor. *Journal of Materials and Environmental Science*.

[34] Dey S, Mukherjee S (2010). Performance and kinetic evaluation of phenol biodegradation by mixed microbial culture in a batch reactor. *International Journal of Water Resources and Environmental Engineering*.

[35] American Public Health Association, American Water Works Association, Water Pollution Control Federation (1998). *Standard Methods for the Examination of Water and Wastewater*.

[36] Furumai H, Kazmi A, Furuya Y, Sasaki K (1999). Modeling long term nutrient removal in a sequencing batch reactor. *Water Research*.

[37] Tremblay A, Tyagi RD, Surampalli RY (1999). Effect of SRT on nutrient removal in SBR system. *Practice Periodical of Hazardous, Toxic, and Radioactive Waste Management*.

[38] Lawrence AW, McCarty PL (1970). Unified basis for biological treatment design and operation. *Journal of the Sanitary Engineering Division*.

[39] Li JP, Healy MG, Zhan XM, Rodgers M (2008). Nutrient removal from slaughterhouse wastewater in an intermittently aerated sequencing batch reactor. *Bioresource Technology*.

[40] Fongsatitkul P, Wareham DG, Elefsiniotis P, Charoensuk P (2011). Treatment of a slaughterhouse wastewater: effect of internal recycle rate on chemical oxygen demand, total Kjeldahl nitrogen and total phosphorus removal. *Environmental Technology*.

[41] Kanimozhi R, Vasudevan N (2013). Effect of organic loading rate on the performance of aerobic SBR treating anaerobically digested distillery wastewater. *Clean Technologies and Environmental Policy*.

[42] Kulikowska D, Klimiuk E (2004). Removal of organics and nitrogen from municipal landfill leachate in two-stage SBR reactors. *Polish Journal of Environmental Studies*.

[43] Doyle J, Watts S, Solley D, Keller J (2001). Exceptionally high-rate nitrification in sequencing batch reactors treating high ammonia landfill leachate. *Water Science and Technology*.

[44] Metcalf and Eddy Inc. (1995). *Wastewater Engineering Treatment Disposal Reuse*.

[45] Barnes DP, Bliss PJ (1983). *Biological Control of Nitrogen in Wastewater Treatment*.

